# *EWSR1::SMAD3*-rearranged fibroblastic tumor: A case with twice recurrence and literature review

**DOI:** 10.3389/fonc.2022.1017310

**Published:** 2022-12-15

**Authors:** Li Yang, Linni Fan, Zhiyong Yin, Yixiong Liu, Danhui Zhao, Zhe Wang, Hong Cheng

**Affiliations:** ^1^ State Key Laboratory of Cancer Biology, Department of Pathology, Xijing Hospital and School of Basic Medicine, Fourth Military Medical University (Air Force Medical University), Xi’an, China; ^2^ Department of Cardiology, Xijing Hospital, Fourth Military Medical University (Air Force Medical University), Xi’an, China

**Keywords:** *EWSR1*, *SMAD3*, fibroblastic tumor, ERG, case report

## Abstract

*EWSR1::SMAD3*-rearranged fibroblastic tumor is a recently described entity that mostly occurs in acral locations. Only 15 cases have been reported in the English literature, with a wide age range and marked female predominance. The most common sites are the foot, followed by the hand and the distal lower leg. There are four cases that recurred locally during 5–120 months of follow-up, with no metastases to date. Herein, we presented a case of *EWSR1::SMAD3*-rearranged fibroblastic tumor that recurred twice in a 20-year-old man. The patient presented with a second recurrent painful nodule in the left plantar of the second toe. Grossly, the lesion was pale solid and well-defined, measuring 9 × 8 × 9 mm in size. Histological examination revealed a monomorphic spindle cell tumor composed of cellular fascicles of bland fibroblasts in a collagenous to myxoid stroma with low mitotic activity, which evoked a wide spectrum of differential diagnoses. Immunohistochemically, the tumor cells were diffusely and strongly positive for ERG while negative for S100, α-SMA, CD34, and other vascular markers. An unbalanced rearrangement of *EWSR1* was demonstrated by fluorescence *in situ* hybridization (FISH), and a gene fusion between *EWSR1 exon 7* and *SMAD3 exon 6* was confirmed by RT-PCR and Sanger sequencing. This case recurred twice within 6 years with no sign of further relapse and metastasis at another 9-month follow-up since the last surgery, indicating that this tumor was benign but prone to local recurrence. Nevertheless, more cases and further studies are needed to better interpret the biological behavior of this new entity.

## Introduction

*EWSR1::SMAD3*-rearranged fibroblastic tumor is currently classified as an emerging entity in the fifth edition of the WHO classification of Tumors of Soft Tissue and Bone (1). Only 15 cases have been reported in the English literature (2–6). The tumor mostly occurs in superficial soft tissue at the extremities, with a marked female predominance. They appear to be benign tumors but are prone to local recurrence. The differential diagnoses are challenging, including a series of benign, borderline, and malignant fibroblastic lesions at acral sites. Herein, we present a male case of *EWSR1::SMAD3*-rearranged fibroblastic tumor with twice recurrence in the left plantar of the second toe. The clinical, histopathological, and molecular characteristics of this tumor along with differential diagnoses are summarized to increase awareness of this new entity among pathologists.

## Case description

**Figure 1:**
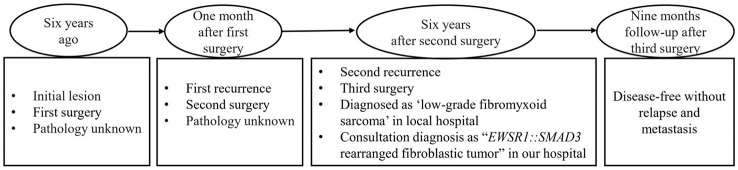
The timeline showed the clinicopathological process.

The patient was a 20-year-old man of Chinese descent presenting with a painful nodule in the left plantar of the second toe for 1 month. For past history, the patient had surgery twice on the same site of the toe 6 years ago and relapsed 1 month later, and there was no other treatment except surgery. Unfortunately, the pathological data were not available, and the operating margin was not known. For this time, the nodule was subcutaneous and palpable. The third surgical resection was performed, and a diagnosis of “low-grade fibromyxoid sarcoma” was made in a local hospital. For further medical attention, the pathological materials were referred to our department for consultation. The clinical follow-up period was 9 months since the last surgery. The timeline is summarized in **Figure 1**.

## Diagnostic assessment

### Clinicopathological characteristics

**Figure 2:**
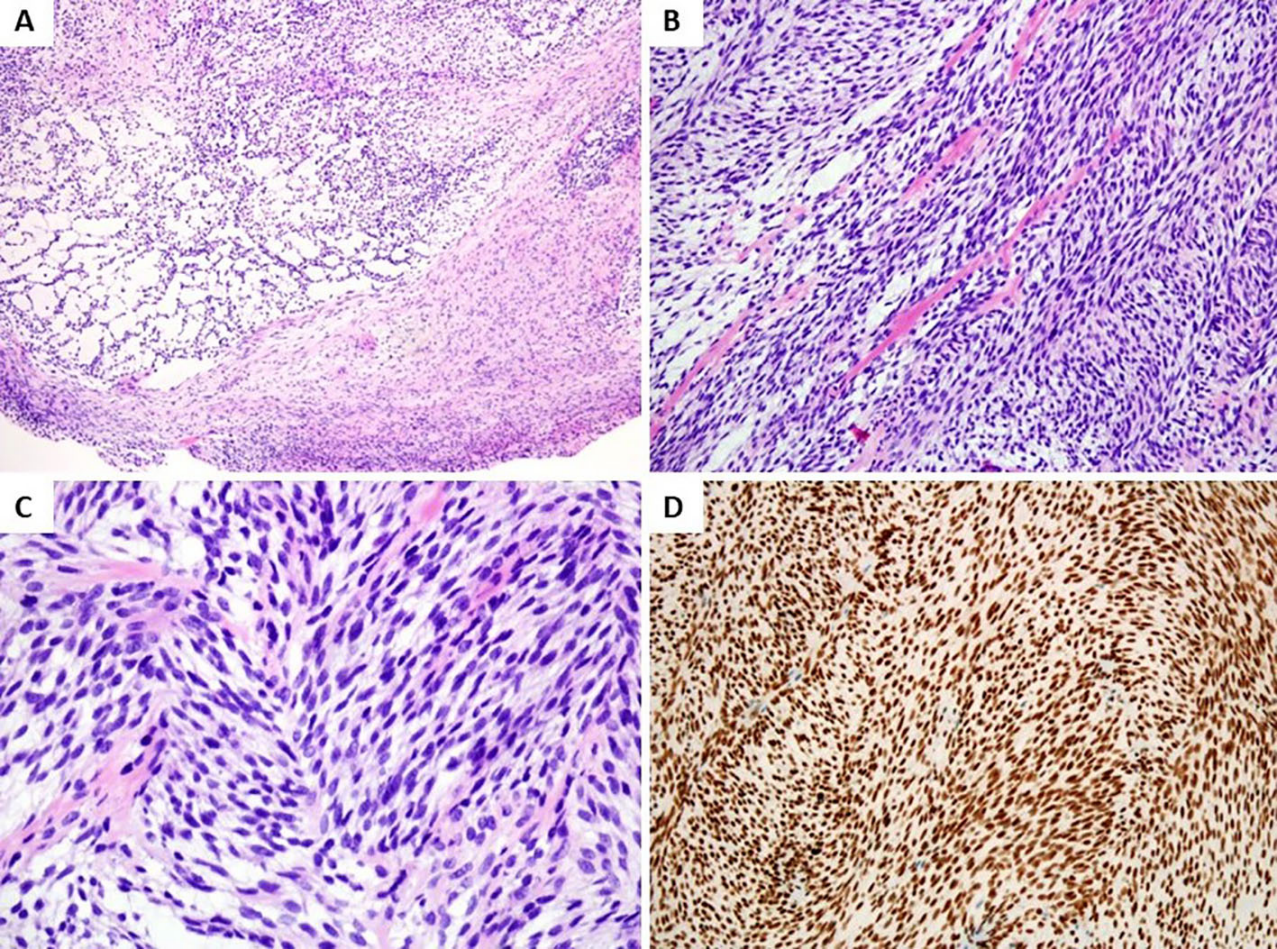
Histologic and immunohistochemical features. **(A)** Histological examination revealed a relatively well-defined, nodular tumor without capsule (H&E, low magnification). **(B)** Monomorphic spindle cell tumor composed of cellular fascicles of bland fibroblasts with hyalinization and mucoid degeneration (H&E, medium magnification). **(C)** Spindle cells showed elongated nuclei with finely dispersed chromatin and small inconspicuous nucleoli (H&E, high magnification). **(D)** Tumor cells showed diffuse nuclear staining for ERG (medium magnification).

Grossly, the lesion was pale solid, and well-defined measuring 9 × 8 × 9 mm in size. Histological examination revealed a relatively well-defined and nodular tumor without a capsule (**Figure 2A**). The tumor was composed of short fascicles of uniform spindle cells in a collagenous to myxoid stroma. The tumor cells had pale eosinophilic cytoplasm and elongated nuclei with finely dispersed chromatin and small inconspicuous nucleoli (**Figures 2B, C**). Mitotic figures were rare.

### Immunohistochemistry

Immunohistochemical staining was performed on 4-µm formalin-fixed, paraffin-embedded sections, which were immersed in a 10-mM sodium citrate buffer (pH 6) for 20 min at 97°C for antigen retrieval. The following antibodies were used: pankeratin (AE1/AE3, 1:50; Dako, Glostrup, Denmark), epithelial membrane antigen (EMA) (E29, 1:200; Dako), bcl-2 (clone 124, 1:100; Dako), CD34 (QB End 10, 1:100; Dako), α-smooth muscle actin (α-SMA) (1A4, 1:200; Dako), desmin (clone D33, 1:200; Dako), S100 protein (polyclonal, 1:800; Dako), SOX10 (polyclonal, 1:200; Santa Cruz Biotechnology, Dallas, TX, USA), H3K27me3 (RM175, 1:2,000, RevMAb, South San Francisco, CA, USA), MUC4 (8G7, 1:100; Abcam, Cambridge, UK), ERG (EPR3864, prediluted; Roche, Basel, Switzerland), SATB2 (SATBA4B10, 1:100, ZSGB), and Ki67 (MIB1, 1:150; Dako).The positive, blank, and negative controls were also performed in parallel.

By immunohistochemistry, the spindle tumor cells showed diffusely and strongly nuclear positive for ERG (**Figure 2D**), while S100, α-SMA, CD34, desmin, SATB2, EMA, MUC4, and other spindle cell tumor and vascular markers were negative. Ki67 index was about 15% in tumors.

### Molecular genetic studies

#### Fluorescence *in situ* hybridization

According to the manufacturer’s instructions, fluorescence *in situ* hybridization (FISH) was performed on formalin-fixed, paraffin-embedded tissue sections. Unstained 4-µm sections were placed on electrostatically charged slides and then evaluated using an *EWSR1* (22q12) rearrangement in a dual-color, break-apart probe and an *EWSR1* (22q12, green) and *SMAD3* (15q22, red) fusion probe (LBP, Guangzhou, China). Under fluorescence microscopy, signals from 200 non-overlapping nuclei were counted. The cutoff level for the score as positive was when at least 20% of the nuclei showed a break-apart signal and/or loss of telomeric part or fusion signal (yellow).

**Figure 3:**
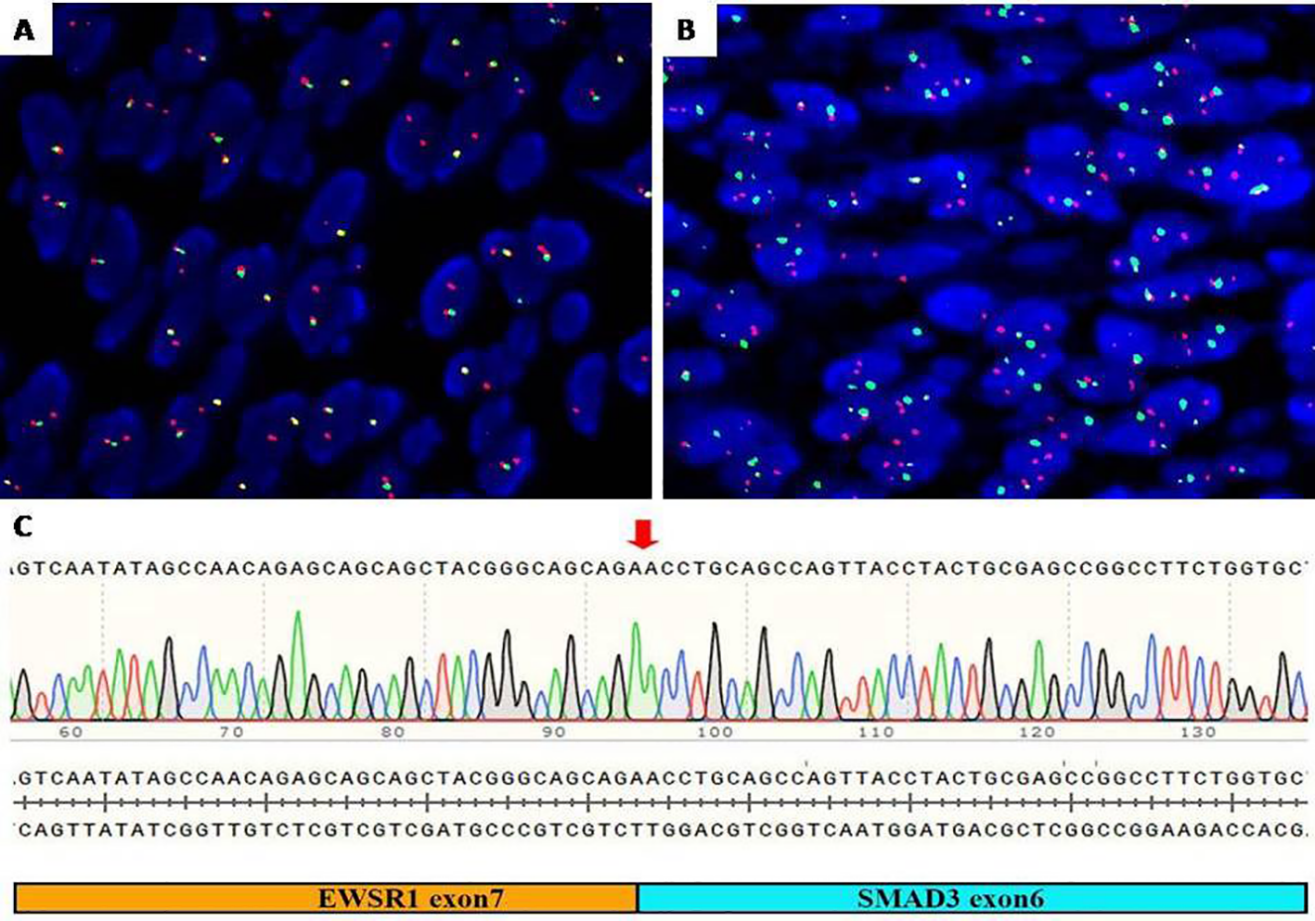
Molecular detection. **(A)** FISH assay showed an unbalanced rearrangement of *EWSR1* with loss of the telomeric part (green). **(B)** FISH assay showed *EWSR1* (green) and *SMAD3* (red) fusion signal (yellow). **(C)** RT-PCR identified *exon 7* of *EWSR1* and *exon 6* of *SMAD3* gene fusion. FISH, fluorescence *in situ* hybridization.

The FISH assessment demonstrated an unbalanced rearrangement of *EWSR1* with loss of the telomeric part (**Figure 3A**), combined with tumor cells, diffuse nuclear staining for ERG the diagnosis of *EWSR1::SMAD3*-rearranged fibroblastic tumor was considered. To identify the fusion partner, *EWSR1* and *SMAD3* fusion was detected by FISH (**Figure 3B**), and the fusion was confirmed by RT-PCR and Sanger sequencing between *exon 7* of *EWSR1* and *exon 6* of *SMAD3* (**Figure 3C**).

#### Follow-up study

Based on the morphology, immunohistochemistry, and gene analysis, the final diagnosis was *EWSR1::SMAD3*-rearranged fibroblastic tumor. Treatment of the tumor was wide excision. To date, the patient is disease-free without relapse and metastasis for 9 months after the last surgery.

## Discussion

*EWSR1::SMAD3*-rearranged fibroblastic tumor is a new entity that mostly occurs at the extremities, initially reported in the foot of a 1-year-old infant, presenting with an ill-defined dermal and subcutaneous nodule (2). Thereafter, another series described similar cases and suggested the name *EWSR1::SMAD3*-rearranged fibroblastic tumor (3). However, only 15 cases have been reported in the English literature to our knowledge, of which the median age was 39 years (range 1–68 years) with a female predilection (10/15, 67%). The most common affected site was the foot (10 cases), and the other three cases were in the hand and two in the distal lower leg. Clinically, all tumors presented as a dermal or subcutaneous nodule or mass ranging in size from 1 to 2 cm (mean, 1.13 cm; median, 1.1 cm). Four cases recurred locally (4/6, 67%) during 5–120 months of follow-up mostly due to incomplete excision, but without metastases to date (2–6). Our case recurred twice within 6 years, indicating that a long-term follow-up needs to be warranted. The series of cases have long-term follow-up information in only seven cases, including what we report in this case, five cases of recurrence, and only two cases without relapse in the follow-up of 7 and 10 years later. The remaining six cases with follow-up information were newly reported, and more cases of follow-up information need to be collected on the biological behavior of this group of tumors. The data are summarized in **Table 1**.

**Table 1:** Clinical features of cases with *EWSR1::SMAD3*-rearranged fibroblastic tumor.

Authors and year	Case	Age (years)/sex	Location	Depth	Size (cm)	Treatment method	Follow-up (time)
Kao et al., 2018 (2)	1	1/M	Heel	Dermis and subcutis	1.0	Surgical resection	LR (14 months)
	2	61/F	Foot	Subcutis	2.0	Surgical resection	NA
	3	58/F	Toe	Dermis and subcutis	1.1	Surgical resection	LR (5 months)
Michal et al., 2018 (3)	4	5/F and 15	Hand-palm	Subcutis	1.2/0.3	Surgical resection, including primary and recurrence	LR (10 years) 18 years
	5	68/F	Interphalangeal joint of the thumb	Subcutis	1.5 × 0.7 × 0.5	Surgical resection	ANED (10 years)
	6	39/F	Calf	Subcutis	1 × 0.5 × 0.5	Surgical resection	ANED (7 years)
	7	34/F	Left foot-dorsal metatarsal aspect	Subcutis	1.1 × 0.8 × 0.5	Surgical resection	Recent case*
Zhao et al., 2019 (4)	8	24/M	Dorsum of the right foot	Subcutis	1 × 0.8 × 0.5	Surgical resection, including primary and recurrence	LR (24 months)
Foot et al., 2020 (5)	9	28/F	The distal phalanx of 2nd toe	Subcutis	1.5	Surgical resection	NA
Habeeb et al., 2021 (6)	10	27/F	Left lower extremity	Mostly superficial dermis	1.7 × 1.3 × 0.8	Surgical resection	Recent case*
	11	35/F	Left 4th/5th finger web space	Deeper dermis	0.7 × 0.3 × 0.2	Surgical resection	Recent case*
	12	45/F	Right plantar forefoot	Mostly superficial and dermal	0.9 × 0. 7 × 0.2	Surgical resection	Recent case*
	13	46/F	Left 4th toe	Superficial dermis	0.3 × 0.2 × 0.2	Surgical resection	Recent case*
	14	54/M	Left foot	Superficial dermis	0.5 × 0.3 × 0.3	Surgical resection	Recent case*
	15	57/F	Left 5th toe	Deep dermis/subcutis	1.4 × 1.2 × 0.6	Surgical resection	Recent case*
	Current case	20/M	Left 2nd toe	Subcutis	0.9 × 0.9 × 0.8	Surgical resection, including primary and recurrence	LR (1 month and 6 years), ANED (9 months)
De Noon et al., 2021 (6)	Bone case	M/44	Right proximal tibia	intramedullary lesion	8	Neoadjuvant chemotherapy and surgical resection	ANED (7 years)

M, male; F, female; LR, local recurrence; NA, not available; ANED, alive without evidence of disease.

*Newly diagnosed cases as of press time.

Recently, another *EWSR1::SMAD3*-rearranged fibroblastic tumor reported in the bone was supported by imaging and histopathological features (7). The tumor was relatively large (8 cm) with aggressive radiological features and initially diagnosed as an unusual cartilaginous tumor, raising the possibility of chondrosarcoma (possibly dedifferentiated or mesenchymal). Histologically, focal cytological atypia and necrosis were observed. Immunohistochemically, the tumor cells showed very focal ERG nuclear expression. The lesion was treated with complete excision and has shown no signs of relapse over a 7-year follow-up period. Although *EWSR1::SMAD3*-rearranged fibroblastic tumor is currently considered benign in soft tissue, the biological behavior of the bone lesion cannot be inferred from a single case (8).

The cellularity and fascicular morphology of *EWSR1::SMAD3*-rearranged fibroblastic tumor may lead to confusion with other histologic mimics of both benign and malignant soft tissue tumors (9), including fibromatosis, cellular schwannoma, low-grade malignant peripheral nerve sheath tumor (MPNST), monophasic synovial sarcoma, and low-grade fibromyxoid sarcoma. This neoplasm was composed of uniform fibroblastic spindle cells without nuclear atypia, pleomorphism, prominent nucleoli, and mitotic activity. In some cases, a distinctive zonation pattern with acellular central hyalinization and peripheral areas of cellular spindle cell fascicles was present, especially in adults. Stippled dystrophic calcification was found in one-part cases (2, 4). In addition, our case also showed an alternative myxoid change of the stroma, which could be misdiagnosed as a low-grade fibromyxoid sarcoma and low-grade MPNST. Immunohistochemically, the tumor characteristically showed diffuse and strong nuclear staining of ERG and focal and weak expression of SATB2 in a small number of cases but was usually negative for S100, SMA, CD34, and other vascular markers, excluding the endothelial, neurogenic, and smooth muscle differentiation of neoplastic cells. The ubiquitous staining of ERG in the tumor is considered to be attributed to the overexpression of ERG, which was revealed by mRNA expression that was significantly upregulated at even higher levels than vascular neoplasms (2, 10).

*SMAD3* is an important signal transducer in the *TGF-β/SMAD* signaling pathway, which is involved in extracellular matrix synthesis by fibroblasts (2, 11). *EWSR1* gene rearrangement has been found in many bone and soft tissue tumors (12, 13), including Ewing or Ewing-like sarcoma, low-grade fibromyxoid sarcoma, and spindle cell rhabdomyosarcoma, and *EWSR1::SMAD3*-rearranged fibroblastic tumor should be added to the list. *EWSR1* gene rearrangement was most common in Ewing or Ewing-like sarcoma; the tumor cells were uniformly small and round and expressed CD99, ERG, NKX2.2, and most differently, the fusion partners with *EWSR1* including *FLI-1*, *ERG*, and other candidate genes. Low-grade fibromyxoid sarcoma may have been an *EWSRI-CREB3L1* fusion, but MUC4 is highly sensitive and specific immunohistochemistry. Spindle cell rhabdomyosarcoma sometimes appeared in *EWSR1-TFCP2* fusion, combined with immunohistochemical markers such as desmin, myogenin, and MyoD1; the diagnosis can be confirmed.

Identification of *EWSR1::SMAD3*-rearranged fibroblastic tumor is critical to provide prognostic information and guide appropriate therapeutic decisions and most importantly avoid overtreatment (9, 14, 15). When encountered with uniform spindle cells in a collagenous to myxoid stroma, diffuse and strong ERG expression will render a clue for *EWSR1::SMAD3*-rearranged fibroblastic tumor. *EWSR1* rearrangement by FISH would help to give a correct interpretation. The data on the morphology, immunohistochemistry, and molecular genetic characteristics are summarized in **Table 2**.

**Table 2:** Pathological and genetic features of cases with *EWSR1::SMAD3*-rearranged fibroblastic tumor.

Authors and year	Case	Histomorphology	Immunohistochemistry					FISH	*EWSR1::SMAD3* Gene fusion	Gene fusion sites
			ERG	CD34	SMA	S100	SATB2			
Kao et al., 2018 (2)	1	Fibroblastic spindle cell	+	−	−	−	NA	Unbalanced rearrangement of SMAD3 and EWSR1	NGS	NA
	2	Fibroblastic spindle cell, central zone collagenized, and focal calcification	+	−	−	−	NA	EWSR1 gene rearrangement	NGS	NA
	3	Fibroblastic spindle cell, central zone collagenized	+	−	−	−	NA	EWSR1 gene rearrangement	NGS	NA
Michal et al., 2018 (3)	4	Spindle cell component located at the periphery and central hyalinization	+	−	−	−	Focal+	NA	NGS	NA
	5	Spindled cells with randomly intermingled hyalinized component	+	−	−	−	Focal+	NA	NGS	NA
	6	Spindled cells with randomly intermingled hyalinized component	+	−	−	−	NA	NA	NA	NA
	7	Spindle cell component located at the periphery and central hyalinization	+	−	−	−	NA	Unbalanced rearrangement of EWSR1	NGS	NA
Zhao et al., 2019 (4)	8	Bland spindle cells, stromal hyalinization, focal stippled calcification	+	−	−	−	Focal+	Unbalanced rearrangement of EWSR1	NGS	EWSR1 exon 7 and SMAD3 exon 6
Foot et al., 2020 (5)	9	Bland spindle cells and collagenous stroma	+	−	Focal+	−	NA	EWSR1 gene rearrangement	NGS	EWSR1 exon 7 and SMAD3 exon 5
Habeeb et al., 2021 (6)	10	Uniform spindled cells in a variably collagenous to myxoid stroma	+	−	−	−	NA	NA	NGS	EWSR1 exon 7 and SMAD3 exon 6
	11	Uniform spindled cells and small hyalinized areas	+	−	Focal+	−	NA	NA	NA	NA
	12	Uniform spindled cells and collagenous to myxoid stroma	+	−	−	−	NA	EWSR1 gene rearrangement	NA	NA
	13	Uniform spindled cells and small collagenous nodules	+	−	−	−	NA	NA	NA	NA
	14	Uniform spindled cells and myopericytomatous pattern focally	+	−	−	−	NA	NA	NGS	EWSR1 exon 7 and SMAD3 exon 6
	15	Uniform spindled cells and central hyalinization	+	−	−	−	NA	NA	NGS	EWSR1 exon 7 and SMAD3 exon 5
	Current case	Bland spindle cells and collagenous or myxoid stroma	+	−	−	−	−	Unbalanced rearrangement of EWSR1	FISH fusion probe and RT-PCR	EWSR1 exon 7 and SMAD3 exon 6
De Noon et al., 2021 (7)	Bone case	Spindle to oval cells and myxo-hyaline to chondroid foci	scattered +	−	−	−	NA	EWSR1 gene rearrangement	Whole-genome sequencing	NA

NA, not available; FISH, fluorescence in situ hybridization; NGS, next-generation sequencing.

In conclusion, *EWSR1::SMAD3*-rearranged fibroblastic tumor should be taken into consideration for the differential diagnosis when encountered with spindle cell lesions in acral sites with strong ERG expression only, and further molecular studies should be employed to confirm the diagnosis. More cases and further studies are needed to further understand the nature of this tumor.

## Data availability statement

The original contributions presented in the study are included in the article/supplementary material. Further inquiries can be directed to the corresponding authors.

## Ethics statement

The studies involving human participants were reviewed and approved by the Institutional Review Board Committee of Xijing Hospital. Written informed consent for participation was not required for this study in accordance with the institutional requirements.

## Author contributions

LY and LF wrote the initial draft of the manuscript. ZY analyzed the clinical aspects of the case. HC and ZW co-designed the study, collected the histopathological and molecular studies, interpreted the findings, and edited the manuscript. YL conducted the FISH analysis and interpreted the findings. DZ performed RT-PCR/direct sequencing. All authors contributed to the article and approved the submitted version.
